# Long-Term Follow-Up With Cardiovascular Magnetic Resonance in 2 Patients With Isolated Left Ventricular Apical Hypoplasia

**DOI:** 10.1016/j.jaccas.2026.109319

**Published:** 2026-07-14

**Authors:** Clifford Isenia, H. Carlijne Hassing, Alina A. Constantinescu, Alexander Hirsch

**Affiliations:** a Department of Cardiology, Thorax Center, Cardiovascular Institute, Erasmus MC, Rotterdam, the Netherlands; b Department of Radiology and Nuclear Medicine, Erasmus MC, Rotterdam, the Netherlands; c Department of Cardiology, Radboud University Medical Center, Nijmegen, the Netherlands

**Keywords:** cardiac magnetic resonance, cardiomyopathy, imaging

## Abstract

**Background:**

Isolated left ventricular apical hypoplasia is a rare congenital cardiac anomaly characterized by a truncated, spherical left ventricle, apical fatty replacement, anomalous origin of the papillary muscles, and variable right-ventricular involvement with a right ventricle that wraps around the left ventricle apex. Most published reports are isolated cases with limited follow-up, leaving long-term outcomes unclear.

**Case Summary:**

We describe 2 patients with isolated left ventricular apical hypoplasia who underwent serial cardiovascular magnetic resonance imaging over more than a decade, from childhood into early adulthood.

**Discussion:**

Both patients demonstrated stable ventricular morphology and myocardial tissue characteristics during follow-up, with preserved or only mildly reduced biventricular systolic function and no evidence of adverse remodeling or clinical deterioration.

**Take-Home Messages:**

Isolated left ventricular apical hypoplasia can remain structurally and functionally stable for more than a decade. Cardiovascular magnetic resonance is essential for diagnosis, long-term surveillance, and risk stratification of this congenital anomaly.

## Visual Summary

**Figure:**
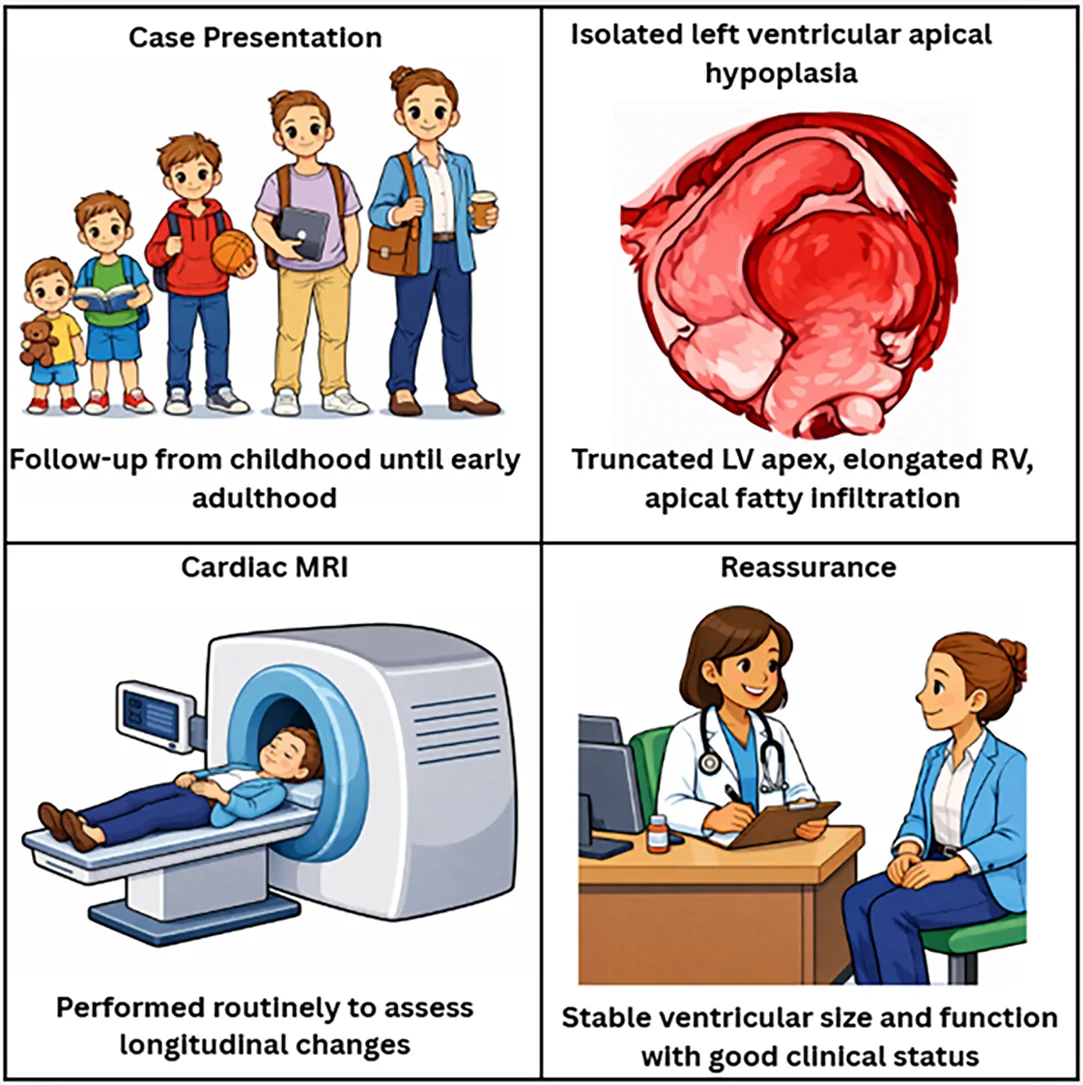

**Visual Summary:**
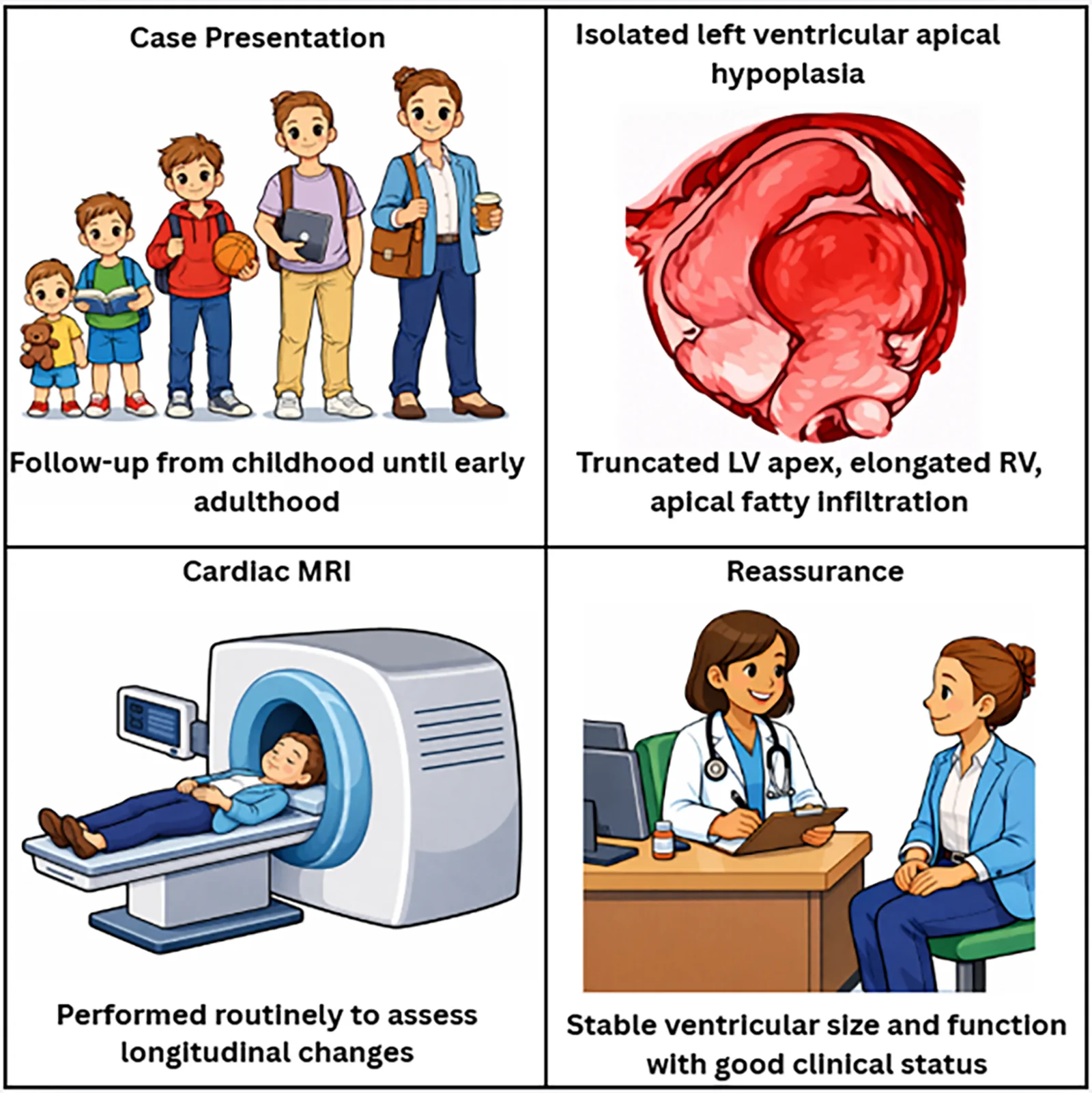
Serial Cardiovascular Magnetic Resonance Imaging in 2 Patients With Isolated Left Ventricular Apical Hypoplasia Demonstrates Long-Term Stability Over More Than a Decade

Isolated left ventricular apical hypoplasia (ILVAH), also known as left ventricular apical hypoplasia or truncated left ventricle, is a very rare congenital cardiac anomaly. It is defined by a non–apex forming left ventricle with a truncated, spherical shape, apical fatty infiltration, anomalous origin of the papillary muscles, and variable ventricular involvement with wrapping of the right ventricle around the deficient left ventricular apex.^1^

Since the first description by Fernández-Valls et al^2^ in 2004, ILVAH has remained exceedingly rare, with only 37 published cases identified in a recent systematic review.^3^ In a comprehensive analysis by Bassareo et al,^3^ the mean age at diagnosis was 26 ± 20 years and the male-to-female ratio was 1.3 to 1. Approximately 35% of patients were asymptomatic, and dyspnea was the most common symptom among symptomatic individuals (41%). Frequent electrocardiographic (ECG) abnormalities included T-wave changes (30%) and right-axis deviation with poor R-wave progression (24%). Atrial fibrillation or flutter was present in 24% of cases, and nonsustained ventricular tachycardia in 8%. A reduced left ventricular ejection fraction (<50%) was observed in 57% of patients, of whom 35% were receiving heart failure therapy. Concomitant congenital anomalies were reported in 16% of cases, most commonly a patent ductus arteriosus. One patient experienced cardiac death.

Overall, ILVAH typically presents in young adults, with variable symptoms and heterogeneous outcomes. Although a substantial proportion of patients presented with preserved ventricular function, more than half of them exhibited left ventricular dysfunction, often accompanied by arrhythmias or symptoms of heart failure. No genetic defect has yet been identified, and associated congenital abnormalities are uncommon, underscoring the persistent gaps in understanding this rare disease.

Although echocardiography may suggest the diagnosis, cardiovascular magnetic resonance (CMR) has become the reference standard owing to its superior assessment of ventricular morphology, function, and myocardial tissue characteristics.

Virtually all published cases are cross-sectional or short-term. No longitudinal data are available on the long-term natural history, structural remodeling, or clinical evolution of ILVAH. In this report, we present 2 young adult patients with ILVAH who underwent serial CMR over more than a decade, capturing progression from early childhood into early adulthood and providing longitudinal insight into this rare cardiac anomaly. These cases offer further insight into ventricular morphology, function, and tissue characterization in this rare congenital anomaly.

## Case Presentation

### Patient 1

#### Patient information and presentation

**Figure 1:**
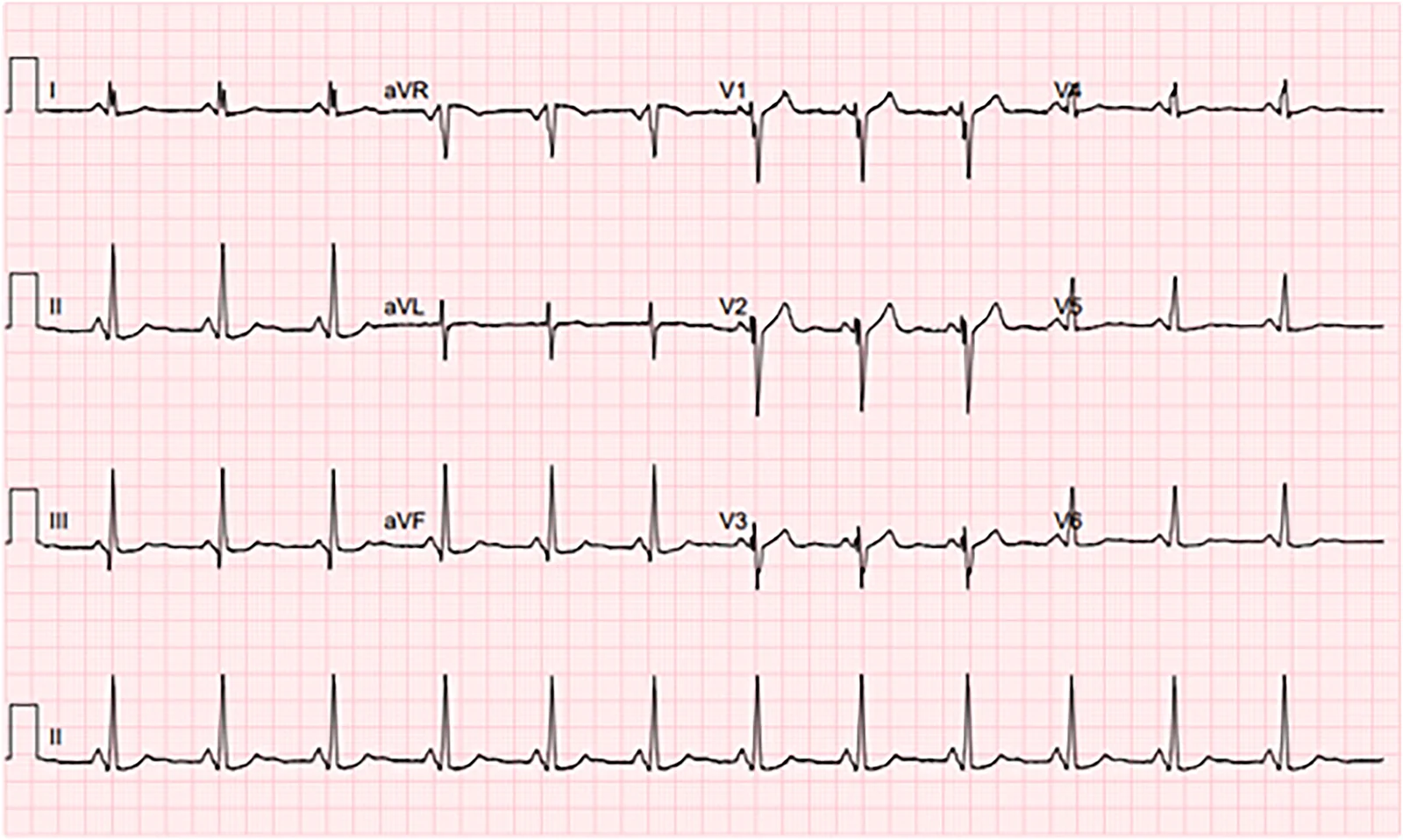
Electrocardiogram at Age 23 in Patient 1 Showing a Slow R-Wave Progression and Nonspecific Repolarization Changes

A 24-year-old man under long-term follow-up for a prenatally diagnosed suspected congenital ventricular abnormality presented for routine cardiac evaluation. Prenatal imaging had revealed a hypoplastic, round-shaped left ventricle. For this reason, he underwent regular cardiology follow-up. CMR performed at the age of 9 confirmed the diagnosis of ILVAH. There was no family history of congenital heart disease. His subsequent childhood was medically uneventful. Physical examination at follow-up was unremarkable, and the current ECG (Figure 1) showed sinus rhythm with a slow R-wave progression on the precordial leads and nonspecific repolarization changes.

#### Cardiovascular magnetic resonance follow-up

**Figure 2:**
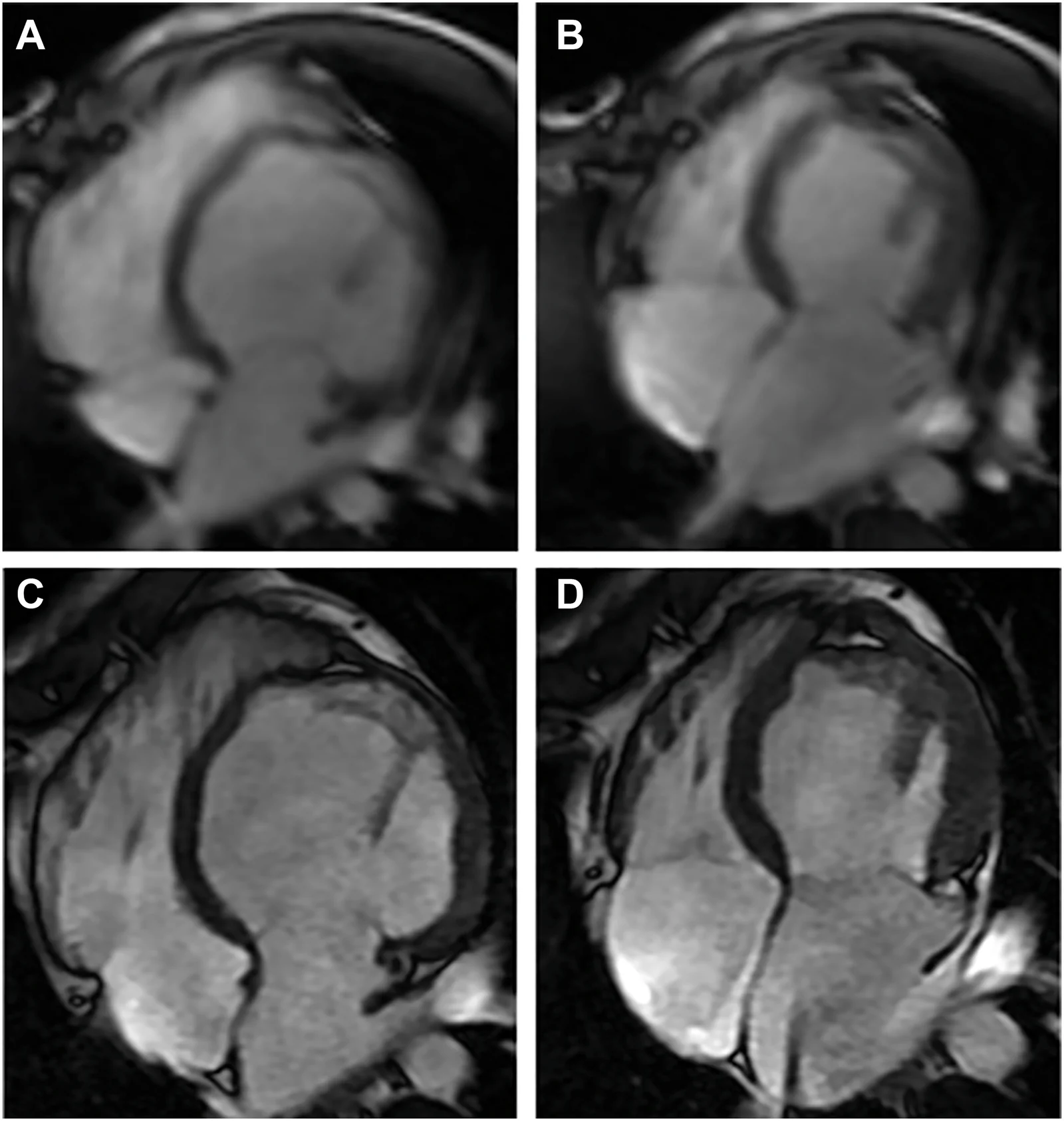
Initial and Most Recent Cardiovascular Magnetic Resonance Performed in Patient 1 at Ages 9 and 23 Cardiovascular magnetic resonance 4-chamber views acquired at end-diastole and end-systole at age 9 (A and B, respectively) and age 23 (C and D, respectively), showing the classic features of isolated left ventricular apical hypoplasia. No morphological changes occurred during follow-up.

**Figure 3:**
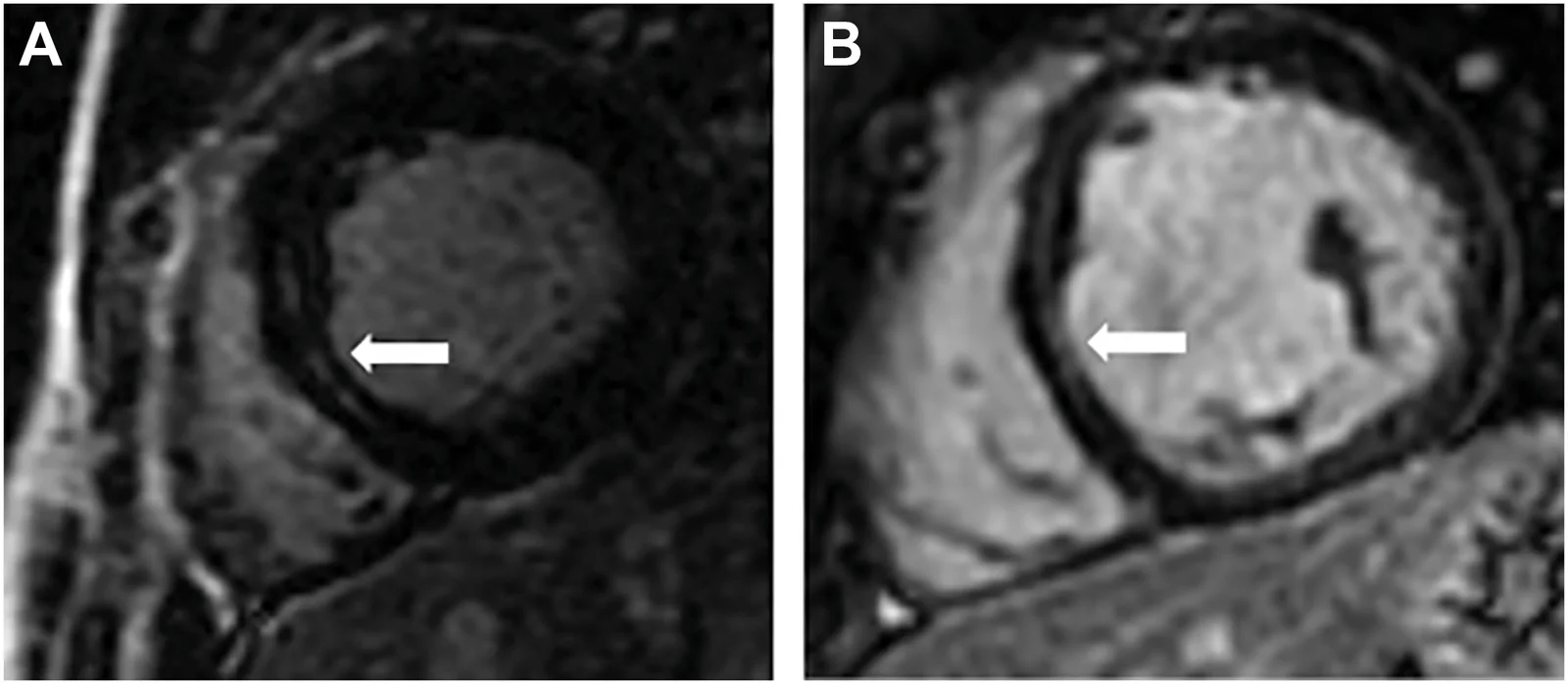
Short-Axis Late Gadolinium Enhancement Images Showing Midwall Septal Late Gadolinium Enhancement in Patient 1 Cardiovascular magnetic resonance (A) at age 9 and (B) age 23. The midwall late gadolinium enhancement was unchanged over the years (white arrow).

The first CMR performed at the age of 9 demonstrated classic features of ILVAH, including a spherical, truncated left ventricular apex, fatty infiltration of the apical region, abnormally inserted papillary muscles, and a right ventricle wrapped around the left ventricular apex (Figure 2). Four-chamber cine images from the most recent CMR examination are also shown. Late gadolinium enhancement (LGE) imaging demonstrated a septal midwall linear pattern of LGE that remained unchanged over 14 years (Figure 3). Table 1 presents the longitudinal follow-up of left and right ventricular volumes and systolic function. The data indicate proportionally stable ventricular volumes with a persistently low-normal left ventricular ejection fraction throughout follow-up.

**Table 1:** Cardiovascular Magnetic Resonance Follow-Up Through the Years in Patient 1

	Age 9	Age 13	Age 17	Age 20	Age 23
BSA (m^2^)	1.03	1.55	1.74	1.69	1.70
LV mass (g)	44	81	96	100	100
LV mass index (g/m^2^)	43	52	55	60	59
LV EDV (mL)	92	178	180	181	182
LV EDV index (mL/m^2^)	89	115	103	108	108
LV ESV index (mL/m^2^)	45	53	51	56	55
LV EF (%)	49	54	51	48	49
RV EDV (mL)	86	169	171	151	150
RV EDV index (mL/m^2^)	83	110	98	89	89
RV ESV index (mL/m^2^)	42	52	47	40	39
RV EF (%)	50	53	52	55	56

BSA = body surface area; EDV = end-diastolic volume; EF = ejection fraction; ESV = end-systolic volume; LV = left ventricular; RV = right ventricular.

#### Management and follow-up

The patient was managed conservatively without medical therapy. Serial echocardiography demonstrated preserved biventricular systolic function and left ventricular diastolic function, without valvular abnormalities. He remained asymptomatic with no signs of heart failure. Twice-yearly Holter monitoring revealed no clinically relevant arrhythmias. Genetic testing, including a comprehensive cardiomyopathy gene panel, was negative. No additional congenital abnormalities were identified.

### Patient 2

#### Patient information and presentation

**Figure 4:**
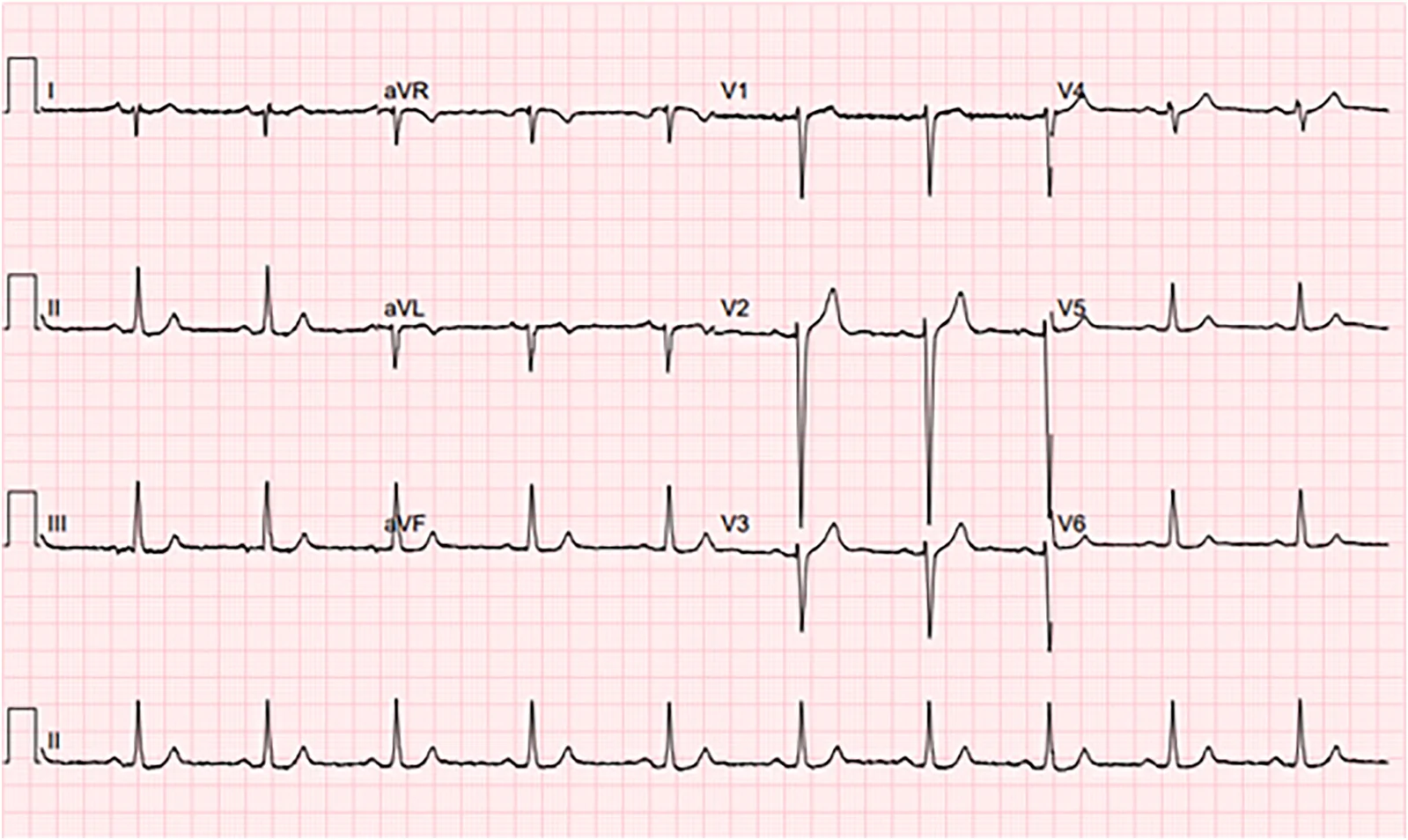
Electrocardiogram at Age 19 in Patient 2 Showing a Right-Axis Deviation and a Slow R-Wave Progression

A 23-year-old man underwent yearly cardiac evaluation starting at age 5. He initially presented with generalized muscle stiffness and pain. His skeletal muscle disease was classified as probable myotonia congenita and was successfully treated with mexiletine. To assess possible cardiac involvement, a referral to a pediatric cardiologist was made. Initial echocardiography demonstrated a round-shaped left ventricle, which was confirmed by CMR, establishing the diagnosis of ILVAH. There was no family history of congenital heart disease. At the latest follow-up at age 23, he had good exercise capacity without complaints. The ECG showed right-axis deviation with slow R-wave progression in the precordial leads (Figure 4).

#### Cardiovascular magnetic resonance follow-up

**Figure 5:**
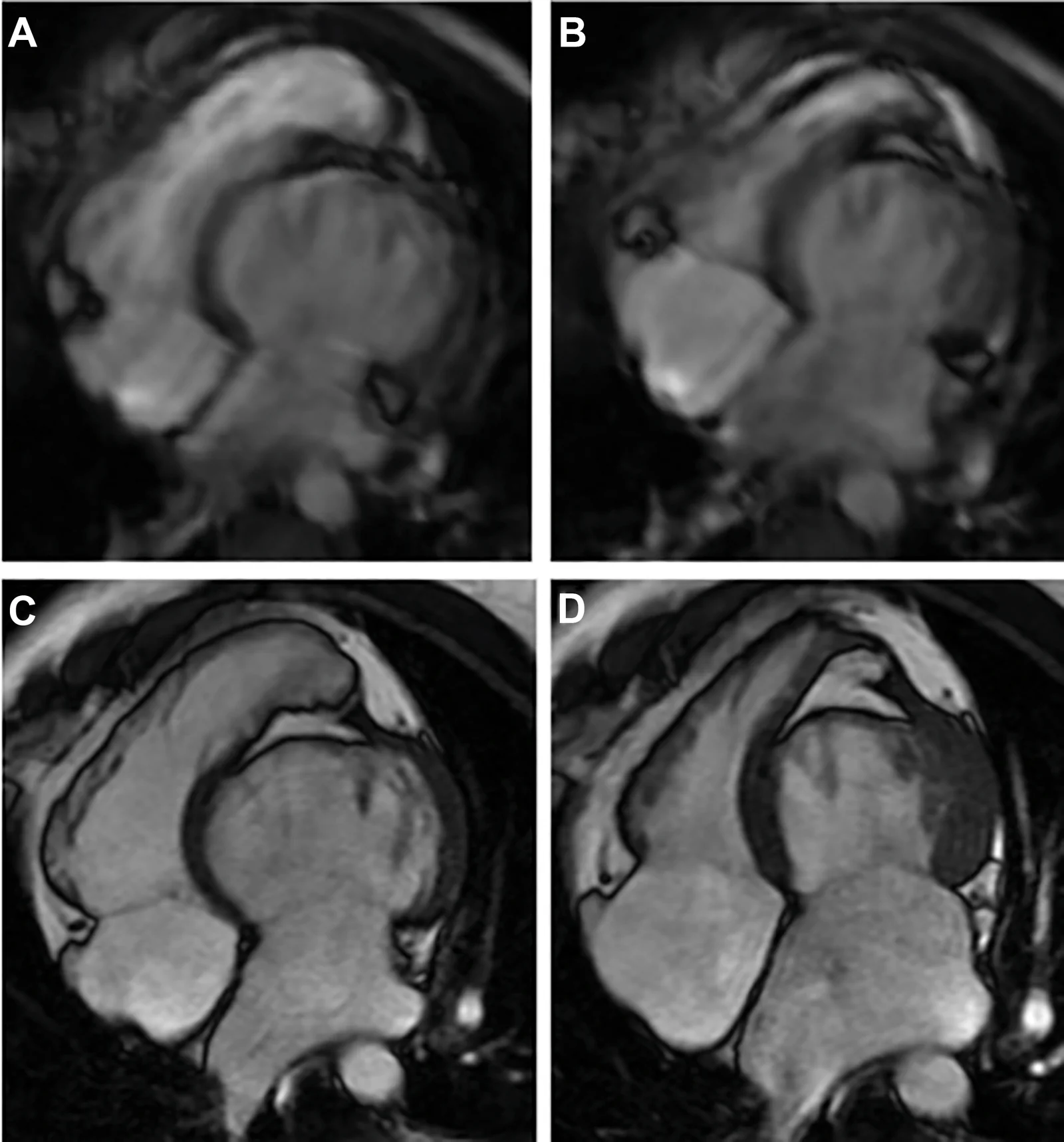
Cardiovascular Magnetic Resonance Performed in Patient 2 at Ages 5 and 19 Cardiovascular magnetic resonance 4-chamber views acquired at end-diastole and end-systole at age 5 (A and B, respectively) and at 19 (C and D, respectively).

**Figure 6:**
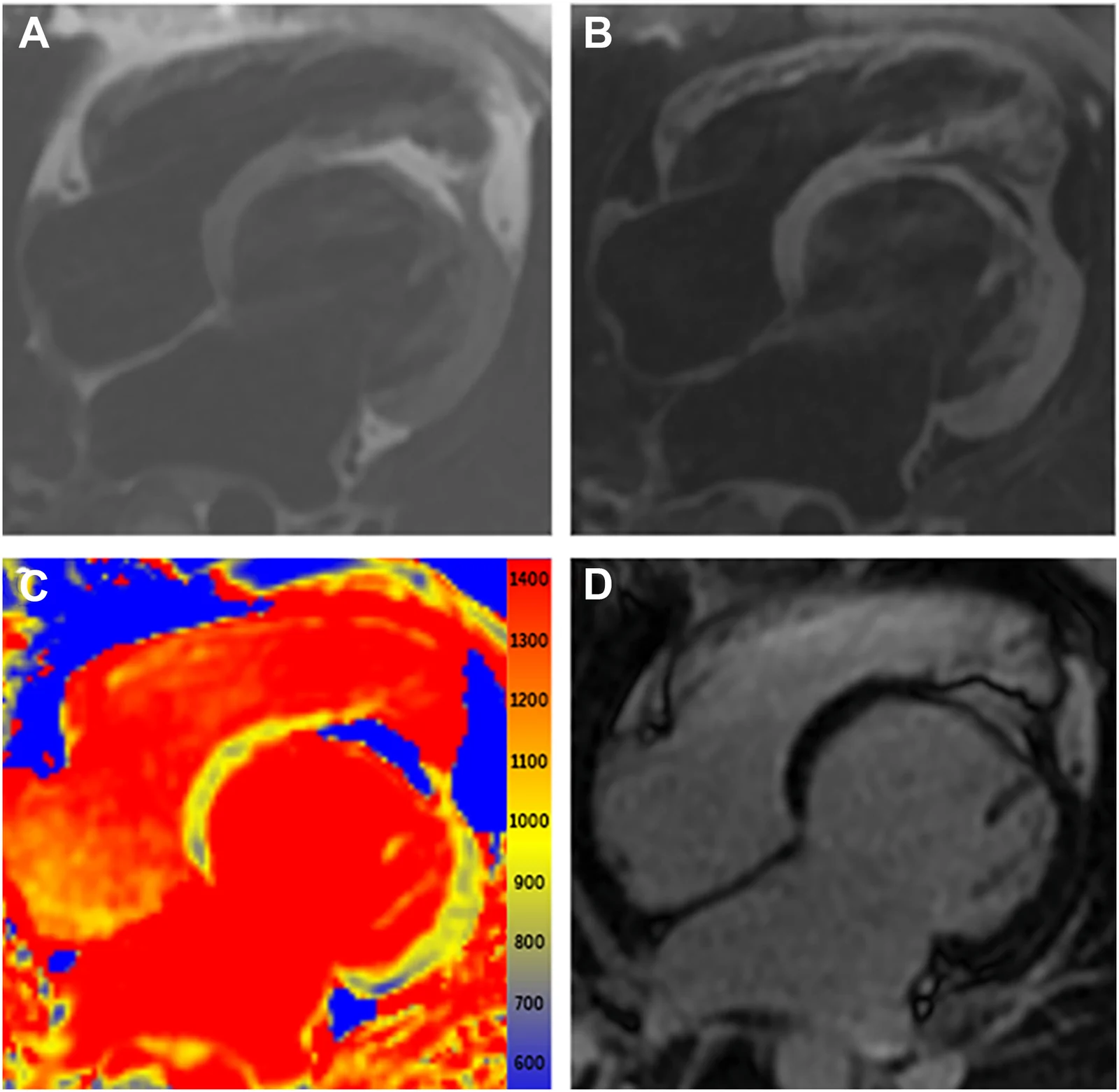
Cardiovascular Magnetic Resonance at Age 19 in Patient 2 With Tissue Characteristics Showing Apical Fatty Infiltration Without Myocardial Late Gadolinium Enhancement (A) T1-weighted image with turbo spin-echo showing a high apical signal suggestive of fatty infiltration. (B) T1-weighted imaging with fat saturation confirming apical fatty infiltration. (C) T1 mapping showing low native T1 values in the apex. (D) Four-chamber view showing no delayed enhancement.

The patient's first CMR was performed at age 5 (Figure 5). The study revealed classical features of ILVAH. The most recent CMR, performed at age 19, is also shown in Figure 5. LGE imaging demonstrated no evidence of fibrosis, which remained unchanged throughout follow-up. On T1-weighted turbo spin-echo images with and without fat suppression, fatty apical infiltration was confirmed. Native T1 mapping revealed low T1 values in the apex, consistent with these findings (Figure 6). Table 2 summarizes longitudinal ventricular volumes and systolic function. Initial evaluation revealed a mildly reduced left ventricular ejection fraction of 43%, which improved gradually during follow-up.

**Table 2:** Cardiovascular Magnetic Resonance Follow-Up Through the Years in Patient 2

	Age 5	Age 10	Age 11	Age 15	Age 19
BSA (m^2^)	0.79	1.24	1.40	1.85	1.90
LV mass (g)	32	46	65	92	85
LV mass index (g/m^2^)	41	37	46	50	45
LV EDV (mL)	75	112	145	184	197
LV EDV index (mL/m^2^)	95	90	103	100	103
LV ESV index (mL/m^2^)	54	37	49	53	49
LV EF (%)	43	59	52	47	53
RV EDV (mL)	54	103	121	167	175
RV EDV index (mL/m^2^)	69	83	86	91	92
RV ESV index (mL/m^2^)	34	27	31	43	40
RV EF (%)	51	68	63	52	56

Abbreviations as in Table 1.

#### Management and follow-up

At initial evaluation at age 5, the patient was started on angiotensin-converting enzyme inhibitor because of mildly reduced left ventricular ejection fraction. At subsequent CMR, left ventricular function was improved and medication was discontinued in 2014 at the patient's request. Serial echocardiographic evaluations demonstrated good systolic and diastolic function of the left ventricle, with no valvular dysfunction. Serial Holter monitoring at intervals of 1 to 2 years revealed no clinically relevant arrhythmias. Serial laboratory testing, including N-terminal pro–B-type natriuretic peptide, remained within normal limits. Genetic testing using a comprehensive cardiomyopathy panel revealed no pathogenic variants. No additional congenital abnormalities were identified.

## Discussion

These 2 cases provide more than a decade of serial CMR follow-up from childhood into early adulthood, offering unique longitudinal insight into the structural and functional behavior of ILVAH. Serial CMR demonstrated remarkable stability of left ventricular morphology, biventricular function, and tissue characterization, with no adverse remodeling. Apical fat infiltration persisted without progression, and LGE, when present, remained unchanged. Clinically, both patients remained asymptomatic, without signs of heart failure or significant arrhythmias. These observations suggest that, at least in some cases, ILVAH may follow a benign long-term course, in contrast to theoretical concerns about progressive ventricular dysfunction or arrhythmic risk. Nonetheless, the lifelong prognosis and optimal management of these patients remain largely unknown. It also remains uncertain which factors predict worse clinical outcomes and whether fibrosis contributes to a higher risk of arrhythmias or other complications.

CMR remains the reference standard for diagnosing and monitoring ILVAH, offering precise evaluation of ventricular volumes, function, and tissue composition. Beyond cine imaging and LGE, parametric mapping for tissue characterization provides valuable diagnostic confirmation and may help differentiate ILVAH from other cardiomyopathies.

These cases underscore the value of long-term serial imaging in establishing prognosis, guiding follow-up, and providing reassurance to patients. Although they contribute valuable insight into the natural history of this rare condition, the conclusions are limited by the small sample size and the unknown trajectory beyond early adulthood. Nevertheless, they represent some of the longest documented follow-ups for this rare congenital phenotype. Multicenter registries and collaborative longitudinal studies will be essential to refine risk stratification and management strategies for this rare congenital condition.

## Conclusions

This case series reports long-term CMR follow-up of ILVAH from childhood into early adulthood. Ventricular morphology, function, and tissue characteristics remained stable, without adverse remodeling or clinical deterioration. These findings suggest a potentially benign course, although larger studies are needed to define long-term prognosis.

## Funding Support and Author Disclosures

Dr Hirsch has received a research grant and consultancy fees from GE Healthcare; speaker fees from GE Healthcare, Bayer, Bristol Myers Squibb, Siemens Healthineers, and Heartflow; is a member of the medical advisory board of Medis Medical Imaging Systems; and was MRI corelab supervisor of Cardialysis BV until 2022. All other authors have reported that they have no relationships relevant to the contents of this paper to disclose.

**Take-Home Messages**

• Isolated left ventricular apical hypoplasia can remain structurally and functionally stable over more than a decade.
• Beyond cine imaging and late gadolinium enhancement, tissue characterization using parametric mapping provides valuable diagnostic confirmation.
• Cardiovascular magnetic resonance is essential for diagnosis, long-term surveillance, and risk stratification in this rare congenital anomaly.
